# Thrombosis of a descending thoracic aortic endovascular stent graft in a patient with factor V Leiden: case report

**DOI:** 10.1186/1749-8090-9-47

**Published:** 2014-03-11

**Authors:** Ganesh S Kumpati, Amit N Patel, David A Bull

**Affiliations:** 1 Division of Cardiothoracic Surgery, University of Utah, 30 N. 1900 E., #3C-127, Salt Lake City, UT 84132, USA

**Keywords:** Endovascular procedures, Descending aorta, Hypercoagulability

## Abstract

We present a case of a 14 year old Caucasian male who underwent initially successful endovascular repair of a traumatic injury to the descending thoracic aorta. The patient had undiagnosed Factor V Leiden at the time of the endovascular repair. He later presented with thrombosis of the endovascular stent graft, necessitating open removal of the stent graft and replacement of the involved aorta with a Dacron graft.

## Background

Endovascular repair for traumatic injury of the descending thoracic aorta is associated with reduced morbidity compared to open repair. As many of the patients undergoing endovascular repair for traumatic aortic disruption are younger, the long term results of endovascular repair in this patient population are unknown. We present a case of a young male who underwent initially successful endovascular repair of a traumatic injury to the descending thoracic aorta. The patient had undiagnosed Factor V Leiden at the time of his endovascular repair. He later presented with thrombosis of the stent graft, necessitating open removal of the stent graft and aortic replacement.

## Case presentation

**Figure 1:**
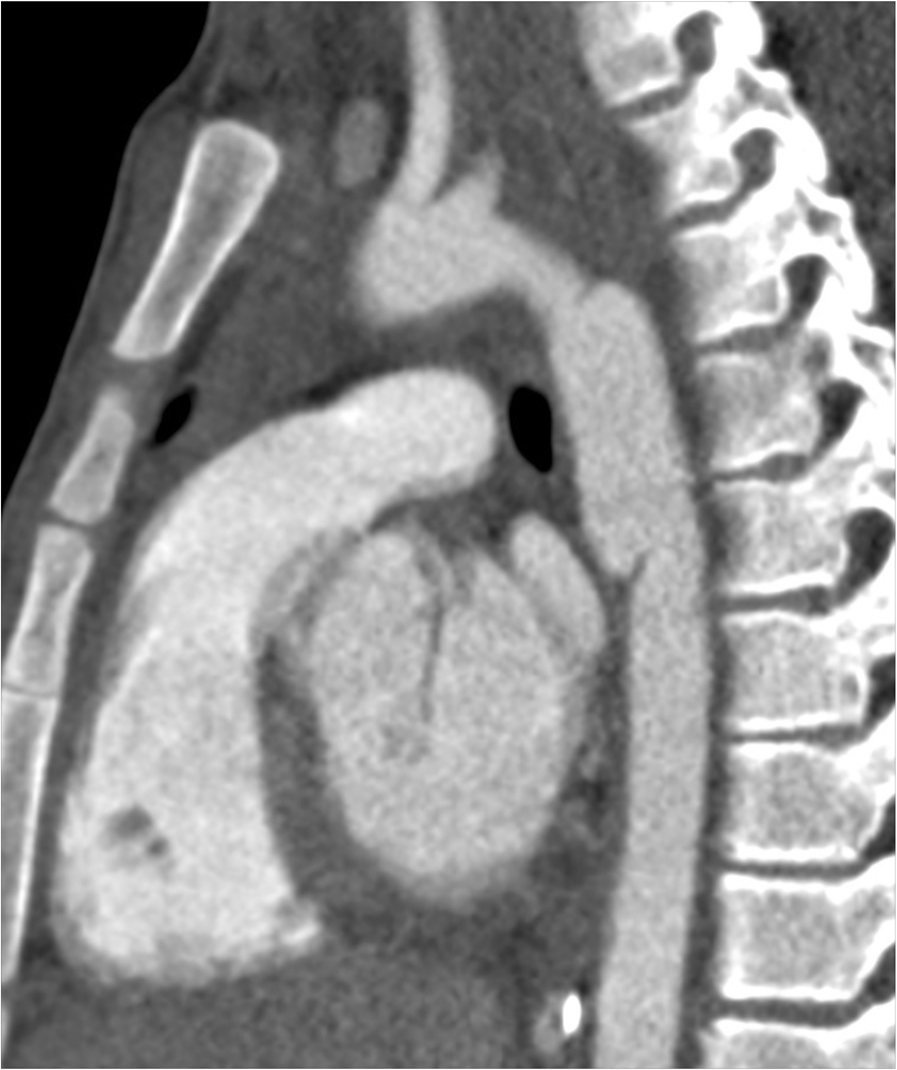
Preoperative CT scan image of traumatic injury to the proximal descending thoracic aorta.

A 14 year old Caucasian male presented after a motor vehicle collision with bilateral rib fractures, pulmonary contusions, a liver laceration, a splenic laceration, a femur fracture, and traumatic disruption of the proximal descending thoracic aorta (Figure 1), with ISS of 29. The patient was neurologically intact (GCS 15) and hemodynamically stable (pulse 90, SBP 100 mmHg) without need for transfusion. He underwent endovascular repair of the thoracic aortic injury 1 day after admission.

**Figure 2:**
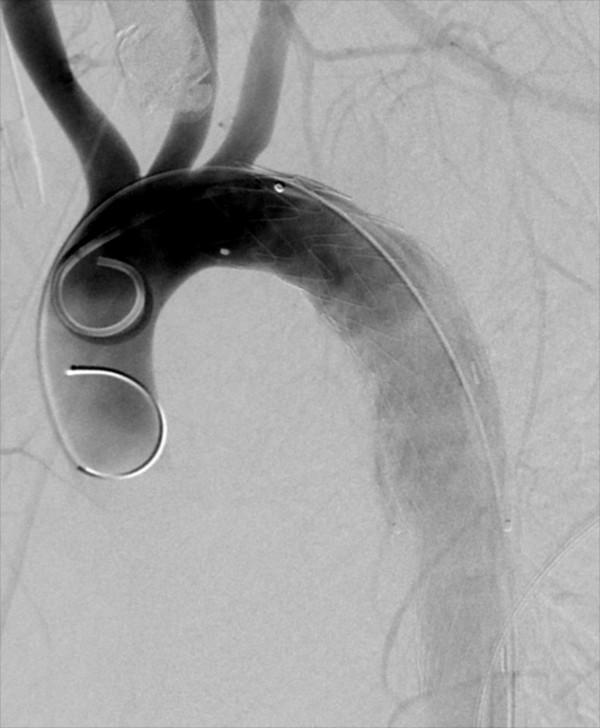
Angiogram image following endograft placement demonstrating seal of aortic injury.

**Figure 3:**
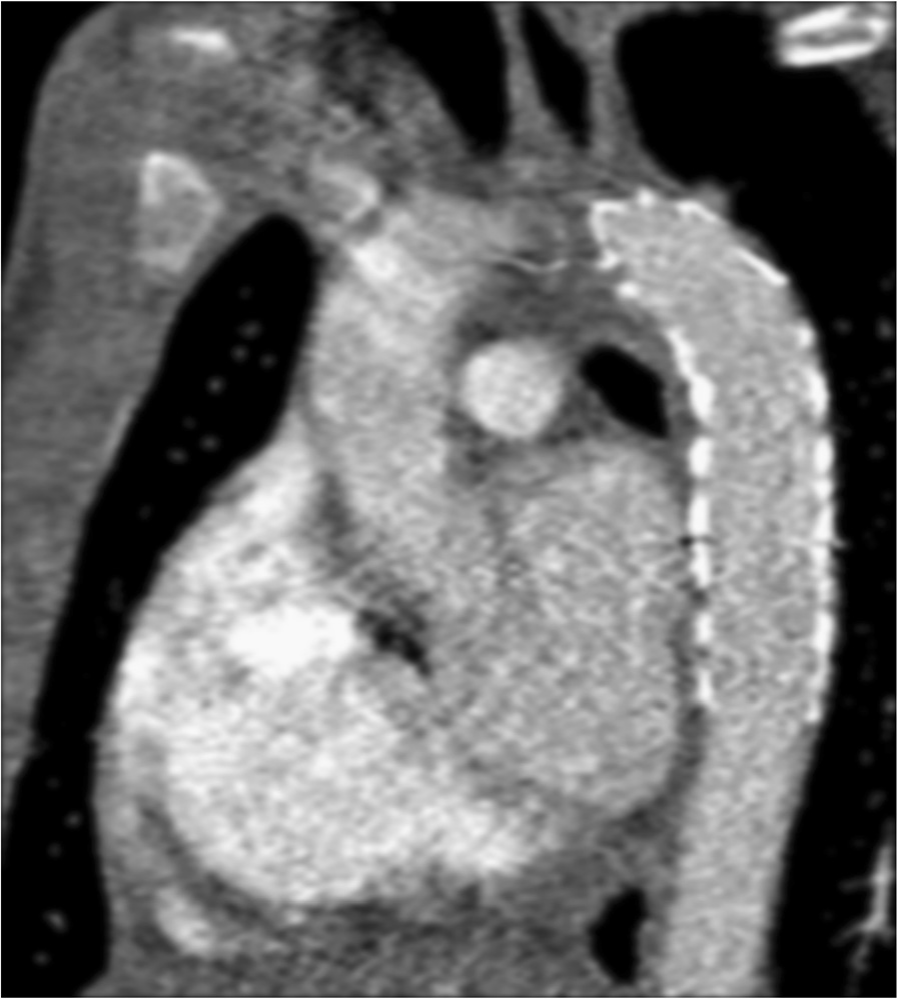
CT image following endograft placement demonstrating seal of aortic injury.

Preoperative imaging demonstrated the proximal and distal aortic diameters to be 16 mm and 14 mm, respectively. Endovascular repair was performed using two overlapping iliac limb devices (Medtronic Endurant 20 mm x 80 mm proximally and Medtronic AneuRx 20 mm x 57 mm as the distal extension) in an off-label indication. The procedure was technically uneventful and post-procedure imaging demonstrated exclusion of the traumatic injury (Figures 2 and 3). He recovered from the other injuries and was discharged to home after a 10 day hospital stay without complications.

**Figure 4:**
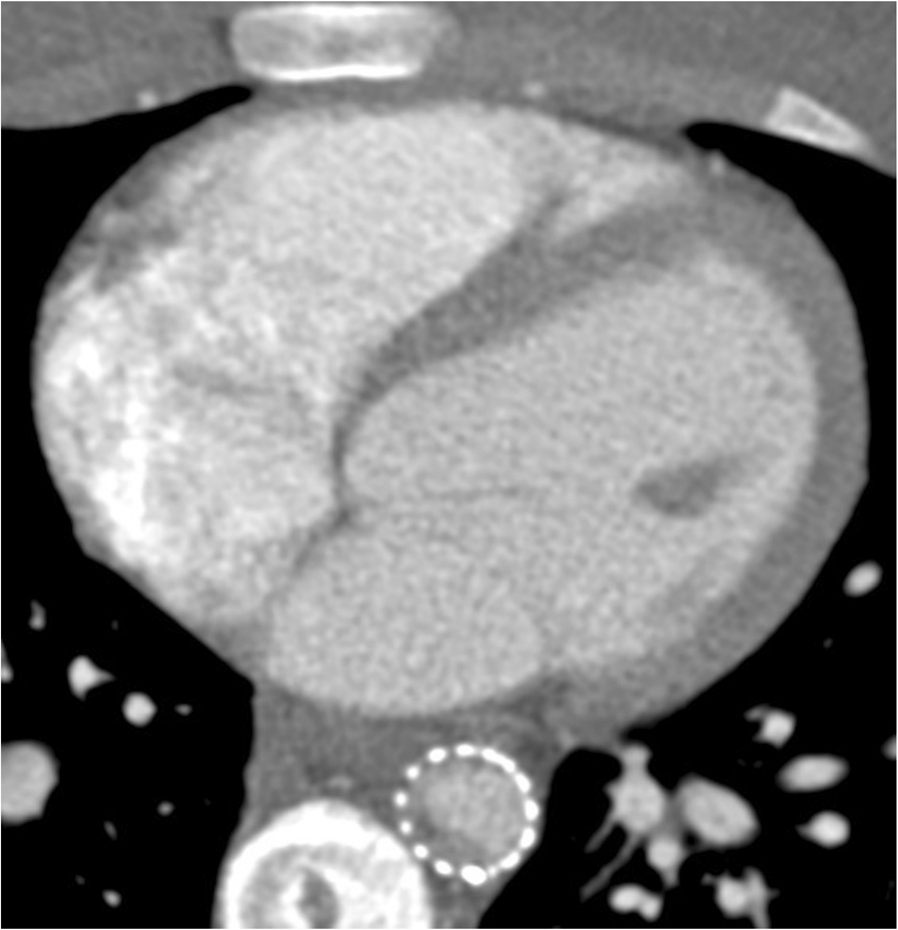
Non-occlusive thrombus within the distal portion of the aortic endograft on initial follow up.

At scheduled follow-up imaging one year later, the CT scan of the thoracic aorta demonstrated non-occlusive intra-luminal thrombus in the distal portion of the endovascular stent graft (Figure 4). There was no thrombus in the native aorta. As part of an evaluation for a hypercoagulable disorder, the patient was diagnosed with Factor V Leiden and placed on oral anticoagulation with warfarin with intention to monitor the thrombus with CT imaging. Follow-up CT imaging demonstrated stable intragraft thrombus.

**Figure 5:**
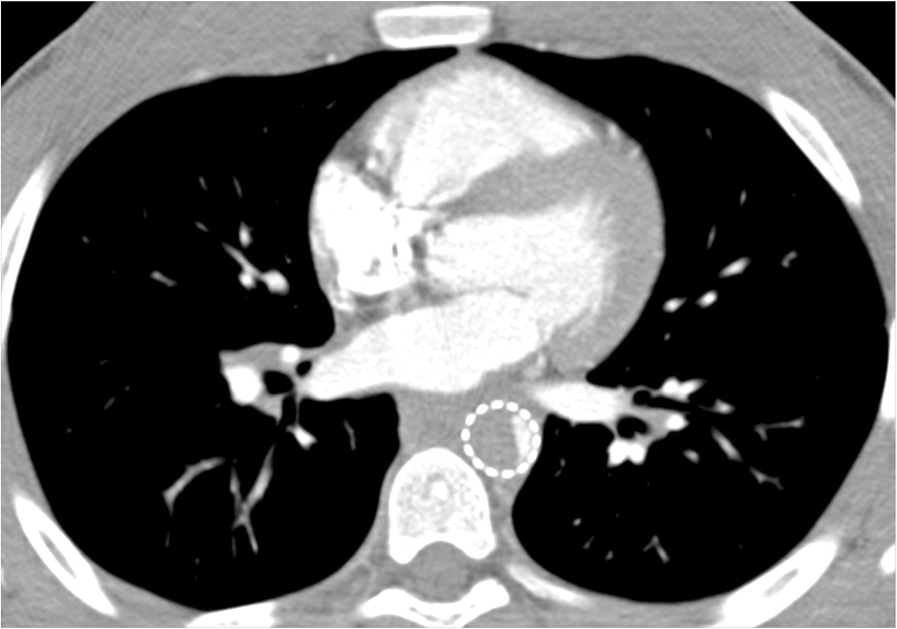
Occlusive thrombus within the distal portion of the aortic endograft following cessation of anticoagulation.

Two years after initial repair, the patient, now 16 years old, stopped his oral anticoagulation after consultation with primary care doctor in order to play competitive sports. Two months after stopping his anticoagulation, he developed progressive lower extremity claudication, which progressed to ischemic rest pain. Examination demonstrated new upper extremity hypertension (brachial SBP 150 mmHg) without palpable pulses in the lower extremities (ankle pressure 68 mmHg, ABI 0.44). A CT scan of the thoracic aorta demonstrated new occlusive thrombus within the distal portion of the stent graft, without any thrombus in the native aorta (Figure 5).

**Figure 6:**
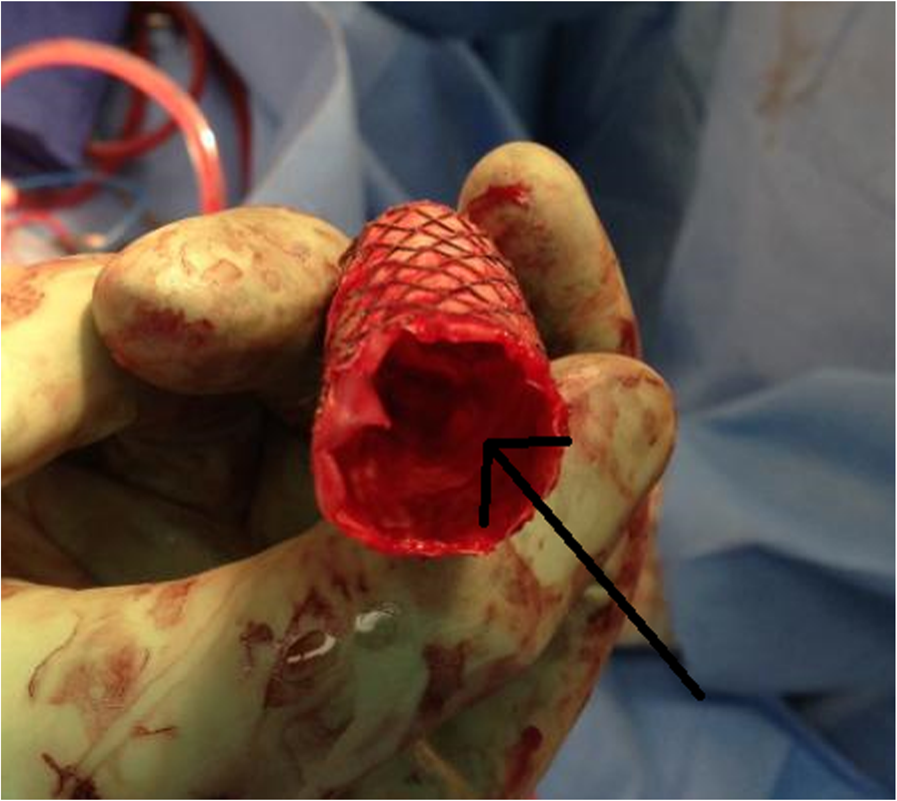
**Proximal aspect of explanted endograft.** (Arrow demonstrating thrombus in the distal portion).

Following a 5 day period of systemic anticoagulation with heparin, the patient did not improve. Therefore, given the presence of severe aortic obstruction due to organized thrombus, we performed open repair via a left thoracotomy. Using distal aortic perfusion, the aorta was cross-clamped between the innominate and left common carotid arteries, and the endograft was removed. The aorta was replaced with a 20 mm Dacron graft from the distal aortic arch, incorporating the origin of the left subclavian artery, to the mid descending aorta. The explanted endovascular stent graft demonstrated solid, organized thrombus in the distal portion with less organized thrombotic material proximally (Figure 6).

**Figure 7:**
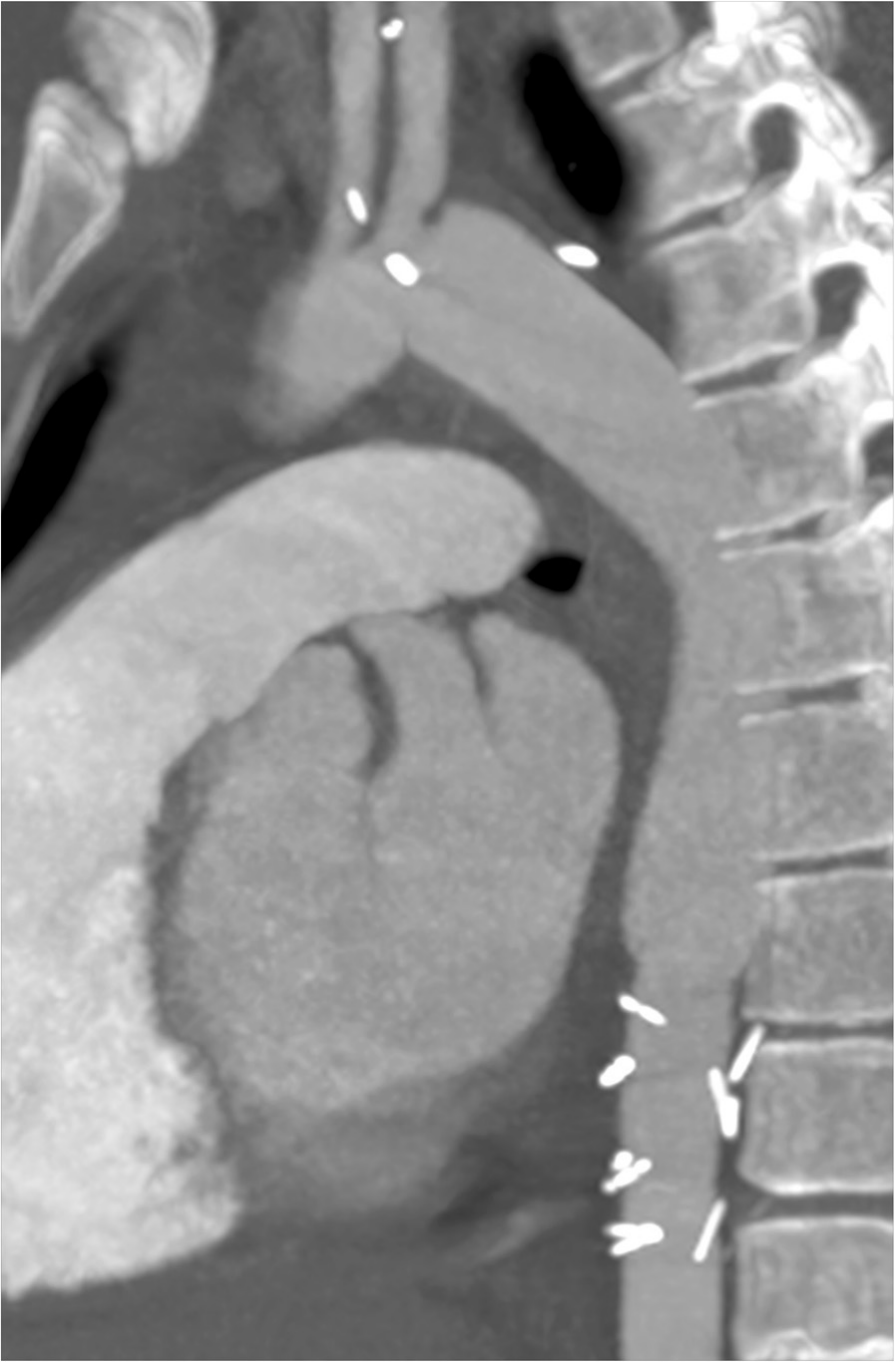
CT scan image following endograft removal and open repair, demonstrating widely patent proximal anastomosis, aortic graft, and distal anastomosis.

Following open repair, the patient had a normal postoperative recovery. The difference in the blood pressures between the upper and lower extremities resolved (brachial SBP 140 mmHg, ankle SBP 150 mmHg, ABI 1.1). The postoperative CT scan demonstrated a normal appearance to the Dacron graft without evidence of thrombus (Figure 7). The patient has been maintained on oral anticoagulation therapy since this surgery, with planned long term anticoagulation therapy. At latest follow up (6 months), the patient is doing well.

## Conclusions

Compared to open repair, endovascular repair has reduced early morbidity for repair of traumatic injury to the descending thoracic aorta. Many of the patients who present with traumatic injury to the descending thoracic aorta are younger patients with normal aortic dimensions. In order to avoid the use of oversized aortic endograft devices, iliac limb endovascular devices with smaller sizes than aortic endovascular grafts are sometimes used in this patient population to achieve a better size match [1,2]. Given the younger age of this patient population, the development of late prosthesis related complications must always be considered.

In-situ thrombosis of thoracic aortic endovascular stent grafts has been described, and has been postulated to be related to infolding or collapse of the endograft [3,4]. Asymptomatic non-occlusive mural thrombus has also been reported within endovascular stent grafts following repair of traumatic aortic disruption [5]. In this case, the role of mechanical flow obstruction in the aorta is unclear given the full graft expansion noted on the initial post procedure imaging.

The Factor V Leiden mutation is a single point mutation in the Factor V gene, which results in a reduced ability of activated protein C to degrade activated Factor V [6,7]. Factor V Leiden is a prothrombotic risk factor, and is associated with an increased incidence of venous thromboembolism, especially when combined with other risk factors for venous thrombosis. The risk of arterial thrombosis is less well defined. Anticoagulation with warfarin is indicated following thrombotic episodes, but the role of prophylactic anticoagulation in the absence of other risk factors is undefined [7].

In this case, the unrecognized hypercoagulable state resulted in the serious complication of occlusive thrombosis of the endovascular stent graft. Consistent, long term therapeutic anticoagulation may help prevent this complication.

## Consent

Written informed consent was obtained from the patient’s legal guardian for publication of this Case report and any accompanying images. A copy of the written consent is available for review by the Editor-in-Chief of this journal.

## Abbreviations

ISS: Injury severity scale; CT: Computed tomography; GCS: Glasgow coma scale; SBP: Systolic blood pressure; ABI: Ankle brachial index.

## Competing interests

The authors declare that they have no competing interests.

## Authors’ contributions

GK drafted the manuscript and developed the concept. AP participated in its design. All authors read and approved the final manuscript.
